# Astronomy from the Moon: the next decades (part 2)

**DOI:** 10.1098/rsta.2023.0079

**Published:** 2024-05-09

**Authors:** Joseph Silk, Ian Crawford, Martin Elvis, John Zarnecki

**Affiliations:** ^1^ Institut d'Astrophysique, Paris, France; ^2^ Department of Physics, The Johns Hopkins University, Baltimore, MD, USA; ^3^ Department of Physics, University of Oxford, Oxford, UK; ^4^ Department of Earth and Planetary Sciences, Birkbeck College, University of London, London, UK; ^5^ Centre for Planetary Sciences at UCL/Birkbeck, Gower Street, London, UK; ^6^ Center for Astrophysics | Harvard and Smithsonian, Cambridge, MA, USA; ^7^ School of Physical Sciences, The Open University, Milton Keynes, UK

There is a dramatic rate of increase in missions to the Moon. Currently, payloads are modest. But in the next decades we will see a dramatic increase in activity as payload costs per kg are forecast to fall dramatically. Lunar telescopes will be a prime beneficiary.^1^ They will lead to compelling and unique science.

Our Royal Society discussion meeting on 13/14 February 2023 sought to review the current renaissance in the development of lunar astronomy projects. It was a sequel to an earlier planned meeting, cancelled because of the COVID epidemic, that did, however, result in publication of a number of articles in a Phil Trans A issue dedicated to Astronomy from the Moon [1,2], in 2021. Here we introduce the sequel, the proceedings of our discussion meeting Astronomy from the Moon: the next decades. The pace of development in those few years is impressive.

Topics over the next decades now include lunar telescopes that cover a wide range of wavelengths, including opportunities for infrared, gravitational wave and cosmic-ray astronomy from the lunar surface.

One major topic is low-frequency radio astronomy from the lunar far side; no fewer than three low-frequency lunar radio astronomy telescopes are scheduled to land in just the next five years [3]. All are exploratory in nature, and designed to test feasibility for future experiments.

The reason for this intense activity is that the sweet spot for observing hydrogen in absorption against the cosmic microwave background (CMB) is at the epoch when the diffuse intergalactic hydrogen medium was cooler than the CMB photons; between a redshift of 30 and 100. The corresponding frequencies of the 21 cm line, from 50 to 15 MHz, are effectively unobservable from Earth. Instead the far side of the Moon is a good place to hunt for this signal: there is no ionosphere on the Moon to scatter incoming low-frequency radio waves, and no terrestrial interference on the far side—it provides the best environment for low-frequency radio astronomy in the inner solar system. Only from there can we hope to probe the building blocks of the galaxies, myriads of clouds of pristine hydrogen, in absorption against the CMB.

In principle, a single dipole is capable of detecting the global shadow of the dark ages. Detection would open a new horizon in astronomy. Attaining even the global signal will require a sophisticated experiment that can endure far more than a single lunar night, or 14 Earth days. Extracting the full information content of this elusive signal from the dark ages may take decades.

The lunar low-frequency radio story began in 2019 with the Chinese National Space Agency Chang'e 4 antenna, the first payload to land on the far side but which suffered from radio interference from the lander. Xuelei Chen [4] (of the National Astronomical Observatory of China) described the current preparation by China to launch its Discovering the Sky at the Longest Wavelengths programme, a flotilla of nine radio satellites with dipole antennae for interferometery, along with a mother satellite for data transmission, flying in formation in selenocentric orbit, along with a mother ship where the data will be transmitted to Earth every two hours. Launch is projected for 2026.

A different approach comes from the US. Behind much of the recent lunar activity is NASA's ongoing Commercial Lunar Payload Services (CLPS) programme, which uses commercially provided launchers and landers. A number of spacecraft providers have signed up to deliver loads to the lunar surface. Several science payloads are scheduled to be launched every year.

In 2024, the Radio wave Observations at the Lunar Surface of the photoElectron Sheath (ROLSES) programme, described by Jack Burns (University of Colorado), is to be launched by Intuitive Machines under the CLPS programme. It will probe lunar-surface radio reflectivity and the surface photoelectron sheath.

Stuart Bale (University of California, Berkeley) described the Lunar Surface Electromagnetics Experiment (LuSEE Night), a NASA-DOE project to launch in December 2025 featuring a single dipole on the far side to deploy in 2026. Long, multi-lunar night integrations are envisaged. The science goal for these two experiments is detection of a global signal from the dark ages.

On a longer timescale, Burns explains how FARSIDE [5] could follow as an interferometer with 128 antennae. These are all pathfinders, to demonstrate technology and feasibility of detection of anisotropic signals from the dark ages. This is the ultimate goal of low radio-frequency lunar cosmology. It is guaranteed science that will potentially revolutionize cosmology by detecting the only robust signal from an initial period of cosmological inflation.

Real progress is anticipated toward this aim under the European Space Agency's (ESA's) ambitious plans to build much larger radio interferometers on the far side [6]. Dutch radio astronomers Leon Koopmans and Hans Klein-Wolt present plans for European projects. One innovative design for antenna deployment is to print many dipoles onto flexible inflatable tubes and lay these out on the lunar surface. ESA's ARGONAUT lander should be able to deploy an initial array of at least 4 × 4 antennae, and up to 32 × 32 antennae based on some new ideas for antennae designs.

Not to be left behind, China is preparing to build its Large-scale Array for Radio Astronomy on the Farside (LARAF), a far side interferometer with 270 dipoles. Deployment of a lander and orbiter via a LM-5 launch is foreseen for the mid-2030s.

A complementary telescope, important for extracting the elusive signal from the dominant radio foregrounds, is under design by NASA's JPL laboratory as an Arecibo-FAST-type behemoth in the form of a 1 km diameter reflector mesh inside a 3 km crater on the far side of the Moon. The focal plane will be suspended from the crater rims. The high resolution is important for removing the signal from many intervening extragalactic radio sources.

The technology for these various projects is challenging but feasible, based on terrestrial experience. The potential science return is compelling. However, to obtain a definitive signal from primordial hydrogen cloud precursors in the dark ages, an interferometer with hundreds of thousands of dipoles will eventually be needed.

This programme is an extension to higher redshift of ongoing studies of cosmic dawn and reionization. Anastasia Fialkov [7] (University of Cambridge) described the emerging horizon in cosmology, from the exploration of cosmic dawn to the dark ages. A pioneering approach aims to combine low-frequency terrestrial radio telescopes, such as NenuFAR, SKA and HERA, with ongoing development of these lunar projects [5].

The lunar surface allows exploration of a much broader frequency range than with radio astronomy alone. The remarkably low lunar seismic activity can be exploited for gravitational wave detectors [8]. Jan Harms (GSSI) proposes [8] a lunar gravitational-wave antenna that will measure gravitational waves from ongoing black hole mergers. Extraordinarily sensitive seismometers will be set up to take advantage of the natural cold (down to 30 K) in permanently shadowed regions near the poles. The seismometer responses allow similar sensitivities to those of the current terrestrial gravitational-wave observatories LIGO and VIRGO at decahertz frequencies, but in the sub-kilohertz range, thereby bridging the frequency gap with the LISA space interferometer, planned for launch in 2036. A lunar gravity-wave observatory would detect the inspiralling of black holes before they merge.

Large infrared telescopes, with capabilities far beyond JWST or ORIGINS sensitivities and wavelength ranges, and unparalleled by any other planned terrestrial or space telescope, may be possible with a simple design. The ESA ARGONAUT transporter would allow installation of a FIR telescope as large as 13 m diameter in a naturally cryogenically cold, permanently shadowed polar crater.

Jean-Pierre Maillard [9] (IAP) argues that the lack of an atmosphere allows a large lunar FIR telescope to exploit new horizons that are unattainable by any planned free flyer. He also notes how the permanently shadowed polar craters offer simplifications over telescopes in free space, e.g. at L2.

Roger Angel [10] (University of Arizona) and Jean Schneider (Meudon) reviewed the prospects for lunar-based optical/IR interferometers. The lack of an atmosphere allows integration times of hours; vastly larger than the millisecond time-scales achieved with terrestrial interferometers combining beams from very large telescopes. Leaders of the ground-based efforts gained a Nobel prize for observations of stellar orbits very near the Galactic Centre that elucidated the mass of the Sag A^*^ supermassive black hole. The stable lunar platform would allow multi-pixel interferometric imaging of exoplanets at near micro-arcsec resolution with an array of telescopes of modest size. Such a facility could dominate future studies not only of supermassive black holes, but also of nearby exoplanets out to hundreds of parsecs, including biosignatures from habitable exoplanet atmospheres.

In the nearer term, observations from the Moon may address a range of important astrobiological topics. Daphne Stam (TU Delft) describes how observing the Earth from the Moon could help prepare for Earth-like exoplanet characterization. Searches for traces of life on remote exoplanets are one of the prime goals in astronomy.

Perhaps the most worrying obstacle to developing lunar telescopes is the abrasive dust that pervades the lunar environment. Mihaly Horanyi [11] (University of Colorado) describes some of these challenges. The meteoroid flux near the poles needs to be evaluated as does the role of electrostatic levitation of dust and deposition in dark craters where telescopes may be sited.

The Moon is a unique location to study the deep space plasma environment as noted by Iannis Dandouras [12] (University of Toulouse). Owing to the absence of a substantial intrinsic magnetic field and of a collisional atmosphere, solar wind and solar energetic particles arrive almost without any deviation or absorption and impact directly on its surface, interacting with the lunar regolith and the tenuous lunar exosphere. These conditions offer unique opportunities to study solar wind and cosmic ray interactions with the surface of a planetary body, avoiding the Earth's magnetosphere.

Lunar rocks and soils also contain a historical record of astrophysical events and processes, in the form of implanted solar-wind particles and cosmogenic nuclei produced by impacting cosmic rays over billions of years. Ian Crawford (Birkbeck) noted that studies of lunar geology, which would be greatly enabled by human explorers returning to the Moon's surface, have the potential to constrain theories of the evolution of the Sun as a star and the Solar System's past interactions with the wider Galaxy [13].

As future projects are seen to proliferate, several of our meeting participants appealed for development of improved international coordination of lunar radio astronomy, the most immediate need, as well as of future activities across the entire electromagnetic spectrum. If we are to succeed in penetrating cosmology's new horizon, the dark ages, it is imperative to begin to coordinate international development of technological demonstrators for low-frequency radio astronomy projects on the far side of the Moon. This could be in the context, for example, of ESA's proposed Astrophysical Lunar Observatory [14].

All of these astronomical applications—spanning low-frequency radio, gravitational waves, optical/infrared telescopes and high energy cosmic rays—rely on the special lunar environment and often, as stressed by Martin Elvis (CfA|Harvard & Smithsonian), on just a few sites in that environment, that make them possible and unique. Aspects of this environment are delicate and will need protection. Alanna Krolikowski [15] (Missouri S&T) recalls the deterioration of terrestrial clear skies that has followed the deployment of the Starlink satellite with adverse implications for projects such as the Rubin telescope. She notes that a proactive agenda is required to protect the lunar environment from optical and radio interference [16] along with other forms of pollution.

Little international coordination has been achieved since the 1967 UN Outer Space Treaty, with 136 signatories. This notably lacks any enforcement strategy. We need to avoid a Wild-West scenario as lunar and cislunar activities proliferate in the next decade.

There will inevitably be competition from human exploration and even commercial activities. The permanently shadowed craters are key targets because they are likely a source of water on the Moon. Mining and science instrumentation will not obviously co-exist compatibly in the same environment. This becomes all the more urgent owing to the imminent exploitation of NASA's Space Launch System (SLS), an expendable vehicle capable of 100 ton deliveries to low-earth orbit, with an availability of some 25% of this payload for lunar delivery. The projected costs of lunar payload deliveries are rapidly dropping via a new generation of large recyclable launchers that include the SpaceX Starship and Blue Origin's New Glenn spacecraft [17].

The diversity of potentially compelling and unique science from these futuristic lunar projects is remarkable. The dark ages will be explored and robust signatures from inflation will be examined. Probing exoplanet atmospheres for traces of extraterrestrial life will surely be aided by the development of large lunar telescopes, albeit in the far future. Current planning for lunar activities includes robotic and human exploration [18]. In the longer term, lunar astronomy may benefit from the infrastructure provided by human exploration, though robotic missions are expected to dominate the renaissance in lunar astronomy projects in the near term.

## Data Availability

This article has no additional data.

## Footnotes

1 See https://www.globalspaceexploration.org/wp-content/isecg/GER_Supplement_Update_2022.pdf.
